# Association of Circulating YKL-40 Levels and *CHI3L1* Variants with the Risk of Spinal Deformity Progression in Adolescent Idiopathic Scoliosis

**DOI:** 10.1038/s41598-019-41191-4

**Published:** 2019-04-05

**Authors:** Dina Nada, Cédric Julien, Pierre H. Rompré, Marie-Yvonne Akoume, Kristen F. Gorman, Mark E. Samuels, Emile Levy, Jason Kost, Dawei Li, Alain Moreau

**Affiliations:** 1 Viscogliosi Laboratory in Molecular Genetics of Musculoskeletal Diseases, Sainte-Justine University Hospital, Research Center, Montreal, QC Canada; 2 Program of Biomedical Sciences, Faculty of Medicine, Université de Montréal, Montreal, QC Canada; 3 Faculty of Dentistry, Université de Montréal, Montreal, QC Canada; 4 Department of Biological Sciences, California State University, Chico, CA USA; 5 Sainte-Justine University Hospital Research Center, Montreal, QC Canada; 6 Department of Medicine, Faculty of Medicine, Université de Montréal, Montreal, QC Canada; 7 Department of Nutrition, Faculty of Medicine, Université de Montréal, Montreal, QC Canada; 8 Department of Microbiology and Molecular Genetics, University of Vermont, Burlington, Vermont, USA; 9 Neuroscience, Behavior, and Health Initiative, University of Vermont, Burlington, Vermont, USA; 10 Department of Biochemistry and Molecular Medicine, Faculty of Medicine, Université de Montréal, Montreal, QC Canada; 11 Department of Stomatology, Faculty of Dentistry, Université de Montréal, Montreal, QC Canada

## Abstract

The cellular and molecular mechanisms underlying spinal deformity progression in adolescent idiopathic scoliosis (AIS) remain poorly understood. In this study, 804 French-Canadian patients and 278 age- and sex-matched controls were enrolled and genotyped for 12 single nucleotide polymorphisms (SNPs) in the chitinase 3-like 1 (*CHI3L1*) gene or its promoter. The plasma YKL-40 levels were determined by ELISA. We showed that elevation of circulating YKL-40 levels was correlated with a reduction of spinal deformity progression risk. We further identified significant associations of multiple *CHI3L1* SNPs and their haplotypes with plasma YKL-40 levels and scoliosis severity as a function of their classification in a specific endophenotype. In the endophenotype FG3 group, we found that patients harboring the haplotype G-G-A-G-G-A (rs880633|rs1538372|rs4950881|rs10399805|rs6691378|rs946261), which presented in 48% of the cases, showed a positive correlation with the plasma YKL-40 levels (*P* = 7.6 × 10^−6^ and coefficient = 36). Conversely, the haplotype A-A-G-G-G-G, which presented in 15% of the analyzed subjects, showed a strong negative association with the plasma YKL-40 levels (*P* = 2 × 10^−9^ and coefficient = −9.56). We found that this haplotype showed the strongest association with AIS patients in endophenotype FG2 (*P* = 9.9 × 10^−6^ and coefficient = −13.53), who more often develop severe scoliosis compared to those classified in the other two endophenotypes. Of note, it showed stronger association in females (*P* = 1.6 × 10^−7^ and coefficient = −10.08) than males (*P* = 0.0021 and coefficient = −9.01). At the functional level, we showed that YKL-40 treatments rescued Gi-coupled receptor signalling dysfunction occurring in primary AIS osteoblasts. Collectively, our findings reveal a novel role for YKL-40 in AIS pathogenesis and a new molecular mechanism interfering with spinal deformity progression.

## Introduction

Idiopathic scoliosis is a prevalent spinal deformity that affects an average of 1–4% of the global pediatric population^1^. It is characterized by an abnormal three-dimensional curvature of the spine with an onset that can occur between birth and sexual maturity. Thus, it has been classified as infantile, juvenile, or adolescent based on when a curve is initiated^2^. Adolescent Idiopathic Scoliosis (AIS) represents the most common form of scoliosis and occurs between the ages of 10 and 15 years, with girls affected more severely than boys^3^. Although the etiology of AIS remains unclear, the fact that the syndrome is influenced by genetic factors has been widely accepted^4,5^. The great phenotypic heterogeneity of AIS, given the multiple genetic loci identified so far^6^, suggest that AIS is more likely to be multifactorial.

The phenotypic complexity and possible genetic heterogeneity have delayed the progress to articulate our understanding of AIS etiology using traditional genetic approaches. Interestingly, our previous works demonstrated that AIS patients present with a distinctive systemic signalling dysfunction for G inhibitory (Gi)-coupled receptors^7,8^. The differential Gi signalling dysfunction among AIS patients allows for their classification into three distinct biological endophenotypes (FG1, FG2, and FG3), based on the maximum Gi signalling response in cells (osteoblasts and other cell types) exposed to Gi-coupled receptor specific stimuli^9^. The use of endophenotypes in complex diseases has the advantage of partitioning the genetic variations, thus providing greater power to detect a genetic effect. “Endophenotype” is a term that was first introduced to genetics in 1966 to describe “microscopic and internal” characteristics while phenotypes describe “obvious and external” characteristics^10,11^. Endophenotypes are heritable traits that are representative of the molecular path from genes to the phenotype^12,13^.

The *CHI3L1* gene encodes for the secretory factor YKL-40. YKL-40 is a member of the family “mammalian chitinase-like proteins,” which correspond to glycoproteins that bind to heparin. YKL-40 was first discovered in 1989 when it was reported to be secreted *in vitro* by MG63 osteosarcoma cell lines in large amounts^14^. YKL-40 is expressed in many tissues and is secreted by several types of solid tumors. The exact function of YKL-40 in normal tissues and in certain pathological conditions remains unknown. YKL-40 acts as a growth factor in cells involved in tissue remodeling. It may have a role in cancer cell proliferation, survival and their ability to invade surrounding tissue^15^. In addition, elevated serum levels of YKL-40 have also been observed in patients with non-malignant diseases of particular contexts of inflammation^16^. Interestingly, elevation of YKL-40 levels has been previously reported in cerebrospinal fluids of adult patients suffering of degenerative spine diseases resulting possibly from damage or stress to the neural/cartilage structure^17^.

The objectives of the present study were to determine whether a differential expression of the *CHI3L1* gene occurs in AIS and to explore the possible contribution of YKL-40 in AIS pathogenesis. We compared the plasma YKL-40 levels between 728 AIS patients and 216 healthy controls, and then sub-classified these AIS patients according to different covariates such as sex, scoliosis severity, and extended our comparisons as a function of one of the three biological endophenotypes previous reported. We further genotyped 12 single nucleotide polymorphisms (SNPs) in the *CHI3L1* gene to test their associations with the plasma levels of YKL-40 and other phenotypes.

## Results

### Clinical and biochemical characteristics

A summary of demographic features, clinical profiles and plasma YKL-40 levels for our French-Canadian cohorts is provided in Table 1. As expected, there were more females in AIS patients than in controls (Fisher’s exact test *P* = 0.001). Plasma YKL-40 levels and genotypes for the 12 *CHI3L1* SNPs were analyzed for 728 patients with AIS and 216 healthy controls after ancestral and relatedness testing. Stratification by scoliosis severity was determined only in the participants who have reached their skeletal maturity at the time of blood collection, which resulted in 132 AIS patients as severe cases (Cobb angle ≥ 40°) and 227 AIS patients as non-severe cases (Cobb angle 10°–39°). Demographic and clinical data for the second cohort of AIS patients (n = 137) and control subjects (n = 51) genotyped by the multiplex polymerase chain reaction are provided in Supplementary Table 1.

**Table 1:** Clinical and biochemical characteristics of participants.

Groups	Females	Males	All Subjects						
	Mean Age (Years)	Mean Cobb Angle (°)	YKL-40 (ng/ml)	Mean Age (Years)	Mean Cobb Angle (°)	YKL-40 (ng/ml)	Mean Age (Years)	Mean Cobb Angle (°)	YKL-40 (ng/ml)
All AIS	13.7 ± 2.2 (6.9–26.0)	28 ± 16 (10–90)	32 ± 20 (3–326)	14.0 ± 2.1 (7.4–18.2)	22 ± 11 (10–72)	39 ± 36 (8–298)	13.8 ± 2.2 (6.9–26.0)	27 ± 16 (10–90)	33 ± 23 (3–326)
	N = 598			N = 112			N = 710		
Endophenotype FG 1	13.5 ± 2.1 (6.9–18.7)	28 ± 15 (10–83)	30 ± 12 (5–69)	14.2 ± 1.6 (10.9–16.7)	27 ± 20 (10–76)	57 ± 62 (14–298)	13.6 ± 2.0 (6.9–18.7)	28 ± 16 (10–83)	34 ± 27 (5–298)
	N = 124			N = 21			N = 145		
EndophenotypeFG2	13.8 ± 2.2 (7.3–19.1)	30 ± 17 (10–89)	32 ± 19 (3–183)	14.0 ± 2.6 (8.7–18.2)	24 ± 12 (10–56)	36 ± 39 (9–228)	13.9 ± 2.2 (7.3–19.1)	29 ± 17 (10–89)	32 ± 22 (3–228)
	N = 229			N = 28			N = 257		
EndophenotypeFG3	13.6 ± 2.2 (7.7–26.0)	26 ± 15 (10–90)	33 ± 24 (4–326)	14.0 ± 2.3 (7.4–18.2)	22 ± 14 (10–66)	33 ± 16 (8–96)	13.7 ± 2.2 (7.4–26.0)	25 ± 14 (10–90)	33 ± 23 (4–326)
	N = 241			N = 60			N = 301		
Healthy Control Subjects	12.5 ± 3.3 (7.1–18.3)	NA	29 ± 13 (8–81)	12.4 ± 3.1 (7.2–17.6)	NA	28 ± 13 (4–90)	12.5 ± 3.2 (7.1–18.3)	NA	29 ± 13 (4–90)
	N = 124			N = 103			N = 227		

### Association of plasma YKL-40 level with AIS biological endophenotype and sex

**Figure 1:**
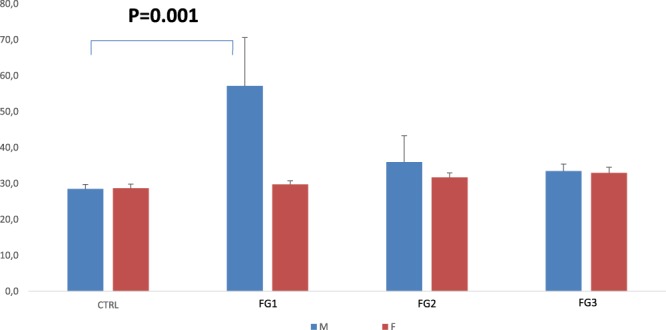
Plasma YKL-40 levels in function of sex and AIS biological endophenotypes. An ANOVA, two-sided T-test was applied and the presented p-values were after Bonferroni adjustment for pair wise comparisons.

Previous work has demonstrated that AIS patients have a distinctive systemic signalling dysfunction for G inhibitory (Gi)-coupled receptors, allowing classifying AIS into three distinct biological endophenotypes with respect to the impedance values: FG1 = [10–40 Ω], FG2 = [40–80 Ω], and FG3 = [80–120 Ω] (while healthy controls always exceed 120 Ω)^8^. This functional classification led us to perform a global expression analysis with primary osteoblasts obtained from AIS patients and trauma cases (as controls). Our data showed a significant overexpression of the *CHI3L1* gene, encoding for the circulating factor YKL-40, in a subgroup of AIS patients (biological endophenotype FG1), which drew our attention given that the AIS patients classified into this endophenotype are less prone to develop a severe scoliosis^18^ when compared to the other two groups (Supplementary Fig. 1). This led us to investigate the possible contribution of YKL-40 in AIS pathogenesis by comparing plasma YKL-40 levels in AIS patients in function of different covariates. We found evidence of a statistically significant interaction between sex and endophenotype (*P* = 0.009). Therefore, we separated the analyses into females and males. By comparing only females, we found no significant differences in circulating YKL-40 levels among the three biological endophenotypes (Fig. 1). While upon analyzing only males, we observed significant differences among the three biological endophenotypes (*P* = 0.001; Fig. 1). After Bonferroni adjustment for pair wise comparisons, the AIS FG1 males (n = 21) showed higher levels than controls males (n = 103) (*P* = 0.001) and AIS FG3 males (n = 60) (*P* = 0.042). Consistently, the changes observed in plasma YKL-40 levels replicated at the protein level our previous expression analyses using primary osteoblasts obtained from AIS patients and matched healthy controls (Supplementary Fig. 1).

### Association of plasma YKL-40 level with scoliosis severity

To assess for possible associations between plasma YKL-40 levels and scoliosis severity phenotype, we classified the AIS patients into severe cases (Cobb angle ≥ 40°) and non-severe cases (Cobb angle 10°–39°). No statistically significant difference was found between the two AIS patient groups or when sex is considered as a covariate. However, we found a statistically significant elevation of plasma YKL-40 levels in the non-severe AIS cases compared to controls (*P* = 0.003).

### Association between plasma Ghrelin and YKL-40 levels

Given the fact that serum YKL-40 levels were previously reported to be inversely correlated with circulating ghrelin levels^19^ and that significantly higher circulating ghrelin levels were previously reported in AIS^20,21^, we measured the plasma ghrelin levels in a subset of our AIS patients and matched healthy controls. The clinical and demographic summary of the participants tested is provided in Supplementary Table 2. Analysis of all AIS patients compared to matched controls showed no significant effect of circulating ghrelin levels on plasma YKL-40 levels. However, when the AIS patients were stratified according to their biological endophenotypes, the mean plasma ghrelin levels were significantly lowered in the FG1 endophenotype samples (99.9 ± 44.9 pg/ml) when compared with the controls (162.8 ± 63.9 pg/ml; *P* = 0.028) and could explain in part the elevation of YKL-40 in this AIS subgroup. In this context, we decided to investigate the 12 SNPs of the *CHI3L1* gene that are known for their regulatory effects on plasma YKL-40 levels in different diseases and healthy populations^14,15,22–25^.

### Associations of the *CHI3L1* variants with plasma YKL-40 levels

To determine whether the *CHI3L1* genotypes affected circulating YKL-40 levels, 12 SNPs were analyzed. Our results showed that eight SNPs were significantly associated with the plasma YKL-40 levels in the AIS patients (Table 2), including rs55700740 (*P* = 3.8 × 10^−5^), rs946259 (*P* = 3.9 × 10^−5^), rs880633 (*P* = 3.8 × 10^−5^), rs1538372 (*P* = 5.0 × 10^−6^), rs4950881 (*P* = 6.0 × 10^−4^), rs946261 (*P* = 4.4 × 10^−8^), rs10920579 (*P* = 1.1 × 10^−8^), and the highest association displayed by rs946262 (*P* = 6 × 10^−12^). By comparison, only two of these SNPs were associated with plasma YKL-40 levels in the healthy controls, including rs1538372 (*P* = 5.7 × 10^−4^) and rs946262 (*P* = 0.0018), which is consistent with the fact that AIS patients showed higher plasma YKL-40 levels than controls (*P* = 0.002).

**Table 2:** Prevalence of the studied SNPs in *CHI3L1* gene and their associations with plasma YKL-40 levels in AIS patients and healthy controls.

SNP	Controls			AIS		
	(N = 216)	Mean YKL-40 (ng/ml) ± SD (95% CI)	P value	(N = 728)	Mean YKL-40 (ng/ml) ± SD (95% CI)	P value
**rs55700740**			0.028			**3**.**85 × 10**^**−5**^
CC	44 (20%)	26 ± 12 (22–30)		167 (23%)	27 ± 12 (25–29)	
CA	111 (51%)	28 ± 11 (26–30)		384 (53%)	34 ± 28 (31–37)	
AA	61 (28%)	31 ± 16 (27–36)		175 (24%)	38 ± 20 (35–42)	
**rs7542294**			0.11			0.952
GG	160 (74%)	28 ± 12 (26–30)		494 (68%)	33 ± 23 (31–35)	
GA	52 (24%)	30 ± 13 (26–34)		212 (29%)	34 ± 27 (30–38)	
AA	3 (1%)	35 ± 24 (8–63)		22 (3%)	32 ± 11 (27–36)	
**rs946259**			0.026			**3**.**87 × 10**^**−5**^
GG	43 (20%)	26 ± 12 (22–30)		164 (23%)	27 ± 12 (25–29)	
GA	109 (51%)	28 ± 11 (26–30)		386 (53%)	34 ± 28 (31–37)	
AA	63 (29%)	31 ± 16 (27–35)		177 (24%)	38 ± 20 (35–41)	
**rs880633**			0.025			**3**.**82 × 10**^**−5**^
GG	63 (29%)	31 ± 16 (35–41)		177 (24%)	31 ± 16 (27–35)	
GA	110 (51%)	27 ± 11 (31–37)		387 (53%)	27 ± 11 (25–29)	
AA	43 (20%)	26 ± 12 (25–29)		164 (22%)	26 ± 12 (22–30)	
**rs1538372**			**0**.**0005**			**5 × 10** ^**−6**^
GG	97 (45%)	31 ± 15 (28–34)		318 (44%)	38 ± 25(35–41)	
GA	98 (45%)	27 ± 10 (25–29)		327 (45%)	31 ± 24 (28–34)	
AA	21 (10%)	22 ± 9 (19–26)		75 (10%)	24 ± 12 (21–27)	
**rs4950881**			0.029			**0**.**0006**
GG	12 (6%)	26 ± 9 (20–31)		48 (7%)	22 ± 12 (19–26)	
GA	80 (37%)	26 ± 9 (21–32)		256 (35%)	32 ± 26 (28–35)	
AA	123 (57%)	30 ± 15 (27–33)		423 (58%)	35 ± 23 (33–38)	
**rs10399805**			0.124			0.523
GG	164 (76%)	28 ± 12 (26–30)		518 (71%)	33 ± 22 (31–35)	
GA	49 (23%)	31 ± 13 (27–35)		189 (26%)	34 ± 28 (30–39)	
AA	2 (1%)	24 ± 3 (20–28)		18 (2%)	33 ± 11 (25–41)	
**rs6691378**			0.107			0.482
GG	166 (77%)	28 ± 12 (26–30)		520 (72%)	33 ± 22 (31–35)	
GA	47 (22%)	30 ± 12 (26–34)		189 (26%)	35 ± 28 (31–39)	
AA	2 (1%)	42 ± 29 (2–82)		18 (2%)	31 ± 9 (27–35)	
**rs946261**			0.036			**4**.**36 × 10**^**−8**^
AA	79 (38%)	30 ± 12 (28–33)		258 (35%)	39 ± 27 (36–43)	
AG	107 (52%)	26 ± 10 (24–28)		355 (49%)	31 ± 23 (29–34)	
GG	21 (10%)	26 ± 14 (20–32)		115 (16%)	25 ± 11(23–27)	
**rs946262**			**0**.**002**			**6 × 10** ^**−12**^
GG	146 (68%)	30 ± 14 (28–32)		477 (66%)	38 ± 27 (35–40)	
GA	65 (30%)	25 ± 9 (23–27)		224 (31%)	26 ± 14 (24–28)	
AA	5 (2%)	19 ± 6 (13–25)		24 (3%)	16 ± 10 (12–20)	
**rs116415868**			0.816			0.645
GG	214 (99%)	28 ± 13 (26–30)		711 (98%)	33 ± 24 (31–35)	
GA	2 (1%)	26 ± 14 (7–45)		16 (2%)	30 ± 8 (26–35)	
**rs10920576**			0.01			**1**.**09 × 10**^**−8**^
GG	158 (74%)	30 ± 14 (27–32)		518 (71%)	36 ± 26 (34–39)	
GA	53 (25%)	25 ± 9 (22–28)		187 (26%)	26 ± 14 (24–28)	
AA	4 (2%)	22 ± 4 (8–36)		22 (3%)	16 ± 10 (12–21)	

*P* ≤ 0.004 is considered significant after Bonferroni correction.

We further divided the patient samples into two groups: non-severe and severe based on their scoliosis phenotype. The same eight SNPs previously associated with plasma YKL-40 levels were still significantly associated with the non-severe cases: rs55700740 (*P* = 0.0006), rs946259 (*P* = 0.0006), rs880633 (*P* = 0.0006), rs1538372 (*P* = 2.0 × 10^−5^), rs4950881 (*P* = 0.0001), rs946261 (*P* = 2.2 × 10^−5^), rs10920579 (*P* = 6.5 × 10^−6^), while the SNP rs946262 still showed the most significant association (*P* = 6.2 × 10^−9^). By comparison, in the severe group, only three SNPs showed marginal associations: rs55700740 (*P* = 0.008), rs946259 (*P* = 0.008), rs880633 (*P* = 0.008) (Table 3).

Similarly, we performed the same analyses separately for each of the three biological endophenotype groups. We found significant associations with plasma YKL-40 levels in the FG2 and FG3 endophenotypes, such as rs946261 (*P* = 0.0001 and *P* = 0.0001, respectively), rs946262 (*P* = 7 × 10^−6^ and *P* = 1.5 × 10^−6^, respectively), and rs10920579 (*P* = 0.0013 and *P* = 2.3 × 10^−5^, respectively). Other SNPs showed specific associations only with AIS patients classified in FG2 endophenotype: rs55700740 (*P* = 0.0005), rs946259 (*P* = 0.0007), rs880633 (*P* = 0.0007), rs1538372 (*P* = 0.0006) (Table 4). Of note, previously reported SNPs rs450928 and rs10399931 known to modulate YKL-40 levels^24,25^ were not included in our initial analysis as they were not assayed in the SNP genotyping array. To address this issue, we performed a targeted sequencing approach (Sanger method) using a limited subgroup of AIS patients (Supplementary Table 3) producing very high circulating YKL-40 levels (>100 ng/ml) and considered as non-severely affected (mean Cobb angle = 21°). Both SNPs were not associated with plasma YKL-40 levels in this subgroup and no additional common or rare variants were detected in *CHI3L1* gene, its proximal promoter and 3′UTR regions among the patients sequenced.

**Table 3:** Prevalence of the studied SNPs in *CHI3L1* gene and their associations with plasma YKL-40 levels in function of scoliosis severity.

SNP	AIS severe (Cobb ≥ 40°)		P value	AIS non-severe (Cobb < 40°)		P value
	N = 132	Mean YKL-40 (ng/ml) ± SD (95% CI)		N = 227	Mean YKL-40 (ng/ml) ± SD (95% CI)	
**rs55700740**			0.008			**0**.**0006**
CC	38 (29%)	29 ± 12 (24–33)		57 (25%)	25 ± 10 (22–28)	
CA	68 (52%)	30 ± 15 (26–34)		107 (47%)	33 ± 14 (30–36)	
AA	26 (20%)	40 ± 12 (35–45)		63 (28%)	39 ± 20 (34–45)	
**rs7542294**			0.651			0.69
GG	93 (70%)	32 ± 15 (29–35)		152 (67%)	33 ± 19 (30–36)	
GA	39 (30%)	30 ± 12 (26–35)		67 (30%)	31 ± 10 (29–34)	
AA	0			8 (4%)	29 ± 7 (25–34)	
**rs946259**			0.008			**0**.**0006**
GG	37 (28%)	28 ± 12 (24–33)		57 (25%)	25 ± 10 (22–28)	
GA	68 (52%)	30 ± 15 (26–34)		106 (47%)	33 ± 14 (30–36)	
AA	27 (20%)	39 ± 12 (34–44)		63 (28%)	39 ± 20 (34–45)	
**rs880633**			0.008			**0**.**0006**
GG	27 (20%)	39 ± 12 (34–44)		63 (28%)	39 ± 20 (34–45)	
GA	68 (52%)	30 ± 15 (26–34)		107 (47%)	33 ± 14 (30–36)	
AA	37 (28%)	28 ± 12 (24–33)		57 (25%)	25 ± 10 (22–28)	
**rs1538372**			0.012			**2 × 10** ^**−5**^
GG	50 (38%)	37 ± 13 (33–41)		100 (45%)	37 ± 16 (34–40)	
GA	59 (45%)	28 ± 14 (24–32)		97 (44%)	31 ± 15 (27–34)	
AA	23 (17%)	29 ± 13 (23–34)		25 (11%)	22 ± 12 (17–27)	
**rs4950881**			0.012			**0**.**0001**
GG	17 (13%)	25 ± 12 (19–31)		15 (7%)	19 ± 11 (13–24)	
GA	52 (39%)	30 ± 15 (25–35)		80 (35%)	30 ± 16 (26–34)	
AA	63 (48%)	35 ± 13 (31–38)		132 (58%)	36 ± 16 (33–39)	
**rs10399805**			0.695			0.353
GG	99 (76%)	31 ± 14 (28–34)		165 (73%)	33 ± 18 (30–36)	
GA	30 (23%)	31 ± 12 (26–37)		56 (25%)	31 ± 10 (28–34)	
AA	1 (1%)	20		6 (3%)	31 ± 7 (25–36)	
**rs6691378**			0.626			0.364
GG	100 (76%)	32 ± 14 (29–35)		165 (73%)	33 ± 18 (30–36)	
GA	30 (23%)	31 ± 12 (26–37)		56 (25%)	31 ± 10 (28–34)	
AA	1 (1%)	20		6 (3%)	31 ± 7 (25–36)	
**rs946261**			0.079			**2**.**2 × 10**^**−5**^
AA	45 (34%)	34 ± 14 (30–39)		87 (38%)	40 ± 20 (36–45)	
AG	67 (51%)	31 ± 14 (27–35)		102 (45%)	29 ± 11 (27–32)	
GG	20 (15%)	27 ± 12 (21–33)		38 (17%)	24 ± 11 (20–28)	
**rs946262**			0.023			**6**.**23 × 10**^**−9**^
GG	78 (60%)	35 ± 13 (31–38)		149 (66%)	37 ± 17 (34–40)	
GA	47 (36%)	28 ± 14 (23–32)		69 (30%)	25 ± 9 (23–28)	
AA	5 (4%)	26 ± 15 (11–42)		8 (4%)	9 ± 4 (8–10)	
**rs116415868**			0.921			0.619
GG	131 (99%)	31 ± 14 (29–34)		223 (98%)	33 ± 16 (30–35)	
GA	1 (1%)	30		4 (2%)	27 ± 14 (10–41)	
**rs10920576**			0.08			**6**.**5 × 10**^**−6**^
GG	85 (64%)	33 ± 13 (30–36)		164 (72%)	36 ± 17 (33–38)	
GA	41 (31%)	29 ± 15 (24–34)		57 (25%)	26 ± 9 (24–29)	
AA	6 (4%)	23 ± 15 (10–37)		6 (3%)	9 ± 4 (5–13)	

*P* ≤ 0.002 is considered significant after Bonferroni correction.

**Table 4:** Prevalence of the studied SNPs in *CHI3L1* gene and their associations with plasma YKL-40 levels in function of AIS biological endophenotypes.

SNP	FG1		P value	FG2		P value	FG3		P value
	(N = 146)	Mean YKL-40 (ng/ml) ± SD (95% CI)		(N = 240)	Mean YKL-40 (ng/ml) ± SD (95% CI)		(N = 286)	Mean YKL-40 (ng/ml) ± SD (95% CI)	
**rs55700740**			0.186			**0**.**0005**			0.035
CC	27 (18%)	28 ± 14 (22–33)		65 (27%)	25 ± 10 (23–28)		66 (23%)	28 ± 14 (25–32)	
CA	78 (53%)	34 ± 34 (26–42)		123 (52%)	33 ± 26 (28–38)		153 (54%)	34 ± 28 (30–39)	
AA	41 (28%)	37 ± 19 (32–43)		50 (21%)	40 ± 24 (33–47)		67 (23%)	37 ± 16 (33–41)	
**rs7542294**			0.613			0.68			0.98
GG	90 (62%)	33 ± 18 (29–36)		170 (71%)	32 ± 22 (29–36)		196 (68%)	34 ± 26 (30–37)	
GA	53 (36%)	36 ± 40 (25–48)		60 (25%)	32 ± 28 (25–39)		81 (28%)	33 ± 14 (30–36)	
AA	3 (2%)	28 ± 8 (19–38)		10 (4%)	29 ± 6 (25–33)		9 (3%)	36 ± 15 (26–46)	
**rs946259**			0.19			**0**.**0007**			0.03
GG	27 (19%)	28 ± 14 (22–33)		65 (27%)	25 ± 10 (23–28)		63 (22%)	28 ± 14 (24–32)	
GA	77 (53%)	34 ± 34 (26–42)		123 (51%)	33 ± 26 (28–38)		156 (54%)	34 ± 27 (30–39)	
AA	41 (28%)	37 ± 19 (32–43)		52 (22%)	40 ± 24 (33–46)		67 (23%)	37 ± 16 (33–41)	
**rs880633**			0.19			**0**.**0007**			0.03
GG	41 (28.1%)	37 ± 19 (32–43		52 (22%)	40 ± 24 (33–46)		67 (23%)	37 ± 16 (33–41)	
GA	78 (53%)	34 ± 34 (26–42)		123 (51%)	33 ± 26 (28–38)		156 (54%)	34 ± 27 (30–39)	
AA	27 (18%)	28 ± 14 (22–33)		65 (27%)	25 ± 10 (23–28)		63 (22%)	28 ± 14 (24–32)	
**rs1538372**			0.045			**0**.**0006**			0.015
GG	79 (54%)	38 ± 34 (30–46)		83 (36%)	38 ± 29 (32–45)		125 (44%)	36 ± 15 (34–39)	
GA	56 (39%)	29 ± 15 (25–33)		123 (53%)	30 ± 19 (27–34)		128 (45%)	33 ± 30 (27–38)	
AA	10 (7%)	26 ± 15 (16–37)		27 (12%)	22 ± 9 (18–26)		33 (12%)	25 ± 14 (20–30)	
**rs4950881**			0.079			0.016			0.091
GG	4 (3%)	17 ± 9 (7–26)		17 (7%)	21 ± 9 (17–26)		23 (8%)	24 ± 14 (18–30)	
GA	51 (35%)	30 ± 17 (26–35)		94 (39%)	30 ± 22 (26–35)		97 (34%)	34 ± 34 (27–41)	
AA	91 (62%)	37 ± 32 (27–44)		129 (54%)	35 ± 25 (31–39)		165 (58%)	34 ± 14 (32–37)	
**rs10399805**			0.436			0.93			0.591
GG	95 (65%)	32 ± 18 (29–36)		177 (74%)	32 ± 22 (29–36)		208 (73%)	33 ± 25 (29–37)	
GA	49 (34%)	38 ± 42 (25–50)		53 (22%)	33 ± 29 (25–41)		69 (24%)	33 ± 14 (30–36)	
AA	2 (1%)	24 ± 3 (20–28)		9 (4%)	28 ± 7 (24–33)		7 (2%)	42 ± 12 (32–52)	
**rs6691378**			0.378			0.972			0.599
GG	95 (65%)	32 ± 18 (26–38)		178 (74%)	32 ± 22 (28–35)		209 (73%)	33 ± 25 (30–37)	
GA	49 (34%)	38 ± 41 (26–50)		52 (22%)	34 ± 29 (26–42)		70 (25%)	34 ± 15 (31–38)	
AA	2 (1%)	24 ± 3 (20–27)		10 (4%)	28 ± 6 (24–32)		6 (2%)	39 ± 10 (30–48)	
**rs946261**			0.133			**0**.**0001**			**0**.**0001**
AA	54 (37%)	36 ± 18 (31–41)		77 (32%)	41 ± 24 (35–6)		106 (37%)	40 ± 33 (34–46)	
AG	70 (48%)	36 ± 36 (27–44)		115 (48%)	29 ± 25 (25–34)		140 (49%)	31 ± 13 (29–33)	
GG	22 (15%)	23 ± 10 (19–28)		48 (20%)	25 ± 10 (22–28)		40 (14%)	25 ± 13 (21–30)	
**rs946262**			0.03			**7 × 10** ^**–6**^			**1**.**5 × 10**^**−6**^
GG	103 (70%)	37 ± 31 (31–43)		138 (58%)	38 ± 26 (33–40)		195 (69%)	38 ± 26 (36–40)	
GA	39 (27%)	28 ± 16 (23–33)		93 (39%)	25 ± 17 (22–29)		79 (28%)	25 ± 9 (23–27)	
AA	4 (3%)	15 ± 8 (6–23)		8 (3%)	15 ± 6 (10–19)		10 (4%)	17 ± 12 (13–21)	
**rs116415868**			0.865			0.977			0.583
GG	145 (99%)	34 ± 28 (29–39)		235 (98%)	32 ± 23 (29–35)		276 (97%)	34 ± 23 (31–36)	
GA	1 (1%)	29.31		5 (2%)	32 ± 8 (26–39)		9 (3%)	29 ± 10 (23–36)	
**rs10920576**			0.036			**0**.**0013**			**2**.**3 × 10**^**−5**^
GG	109 (75%)	36 ± 30 (31–42)		162 (68%)	35 ± 25 (31–39)		206 (72%)	37 ± 25 (33–40)	
GA	33 (23%)	28 ± 16 (22–33)		71 (30%)	26 ± 18 (22–31)		70 (25%)	25 ± 9 (23–27)	
AA	4 (3%)	145 ± 8 (6–23)		7 (3%)	15 ± 6 (10–20)		9 (3%)	18 ± 13 (9–26)	

*P* ≤ 0.001 is considered significant after Bonferroni correction.

### Associations of the *CHI3L1* variants with scoliosis severity

None of the individual SNPs showed a significant association with the disease when AIS cases were compared to the matched healthy control group independently of plasma YKL-40 levels (P > 0.05). However, rs1538372 was the only SNP showing a significant difference when AIS biological endophenotypes were compared. Indeed, this SNP was more strongly associated with AIS patients classified in endophenotype FG1 when compared to AIS cases classified in FG2 after Bonferroni correction (*P* = 0.001) (Supplementary Table 4). Neither did any of the individual SNPs showed any significant difference in function of scoliosis severity. However, when separated by sex, two SNPs showed significant differences between the severe AIS patients and healthy controls in the males: rs946262 and rs10920579 (*P* = 0.012 and *P* = 0.005 respectively).

### Haplotype analysis of the *CHI3L1* variants

**Figure 2:**
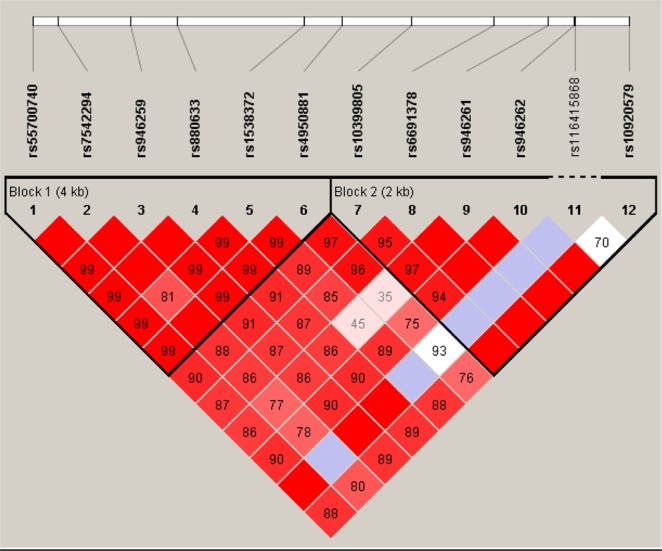
Linkage disequilibrium blocks of the 12 SNPs tested in *CHI3L1* gene.

Strong linkage disequilibrium was found among the selected 12 SNPs, as shown in Fig. 2. We, therefore, investigated whether certain haplotypes were associated with the plasma YKL-40 levels. We found evidence of strong associations of haplotypes with the plasma YKL-40 levels (Table 5). For instance, the haplotype A-A-G-G-G-G (rs880633|rs1538372|rs4950881|rs10399805|rs6691378|rs946261), which presented in 15% of the analyzed subjects, showed a strong negative association with the plasma YKL-40 levels (*P* = 2 × 10^−9^ and coefficient = −9.56). By further stratifying the samples into three biological endophenotypes (sample sizes shown in Supplementary Table 5), we found that this haplotype showed the strongest association in endophenotype FG2 (*P* = 9.9 × 10^−6^ and coefficient = −13.53), compared to endophenotypes FG1 (*P* = 0.0044 and coefficient = −11.42) and FG3 (*P* = 0.031 and coefficient = −19.14) (Tables 6, 7, and 8). Of note, it showed stronger association in females (*P* = 1.6 × 10^−7^ and coefficient = −10.08) than males (*P* = 0.0021 and coefficient = −9.01) (Tables 9 and 10). In the endophenotype FG3 group, we also found the haplotype G-G-A-G-G-A (rs880633|rs1538372|rs4950881|rs10399805|rs6691378|rs946261), which presented in 48% of the cases, showed positive correlation with the plasma YKL-40 levels (*P* = 7.6 × 10^−6^ and coefficient = 36; Table 8). In females, we found another haplotype A-A-G-G-G-G (rs10399805|rs6691378|rs946261|rs946262|rs116415868|rs10920579) that showed significant association with plasma YKL-40 levels (*P* = 2.8 × 10^−6^ and coefficient = −8.43; Table 9). All these results support strong association of haplotypes with plasma YKL-40 levels in relation of the risk of disease progression.

**Table 5:** Haplotype association analyses of plasma YKL-40 levels

SNP IDs	SNPs	Haplotype	Freq	Coeff	Std. Error	Pr(>|t|)
rs55700740|rs7542294|rs946259|rs880633|rs1538372|rs4950881	1-2-3-4-5-6	C-G-G-A-A-A	0.074	−5.93	2.09	**0**.**0047**
rs7542294|rs946259|rs880633|rs1538372|rs4950881|rs10399805	2-3-4-5-6-7	G-G-A-A-A-G	0.069	−5.13	2.12	**0**.**016**
rs946259|rs880633|rs1538372|rs4950881|rs10399805|rs6691378	3-4-5-6-7-8	G-A-A-A-G-G	0.085	−4.92	1.99	**0**.**014**
rs55700740|rs7542294|rs946259|rs880633|rs1538372|rs4950881	1-2-3-4-5-6	C-G-G-A-A-G	0.242	−4.6	1.66	**0**.**0056**
rs7542294|rs946259|rs880633|rs1538372|rs4950881|rs10399805	2-3-4-5-6-7	G-G-A-A-G-G	0.242	−4.12	1.65	**0**.**013**
rs946259|rs880633|rs1538372|rs4950881|rs10399805|rs6691378	3-4-5-6-7-8	G-A-A-G-G-G	0.242		1.66	0.025
rs880633|rs1538372|rs4950881|rs10399805|rs6691378|rs946261	4-5-6-7−8-9	A-A-G-G-G-G	0.151	−9.56	1.58	**2**.**4 × 10**^**−9**^
rs946259|rs880633|rs1538372|rs4950881|rs10399805|rs6691378	3-4-5-6-7-8	A-G-G-A-G-G	0.505		1.94	0.0317
rs1538372|rs4950881|rs10399805|rs6691378|rs946261|rs946262	5-6-7-8-9-10	G-A-A-A-G-G	0.134	−5.28	1.86	**0**.**0048**
rs4950881|rs10399805|rs6691378|rs946261|rs946262|rs116415868	6-7-8-9-10-11	A-A-A-G-G-G	0.139	−5.44	1.98	**0**.**0062**
rs880633|rs1538372|rs4950881|rs10399805|rs6691378|rs946261	4-5-6-7-8-9	A-A-G-G-G-A	0.090		1.83	0.020
rs10399805|rs6691378|rs946261|rs946262|rs116415868|rs10920579	7-8-9-10-11-12	G-G-A-G-G-G	0.600		3.54	0.025

823 samples, including 631 cases and 194 controls, were analyzed. Mean age = 13.38. Regression analysis was used. Only *P* values < 0.05 are shown. *P* values < 0.017 (Bonferroni correction threshold) are in bold. Freq = haplotype frequency; Coeff = coefficient (only shown when corrected *P* values < 0.05).

**Table 6:** Haplotype association analyses of plasma YKL-40 levels in endophenotype 1.

SNP IDs	SNPs	Haplotype	Freq	Coeff	Std. Error	Pr(>|t|)
rs880633|rs1538372|rs4950881|rs10399805|rs6691378|rs946261	4-5-6-7-8-9	A-A-G-G-G-G	0.117	−11.42	3.93	**0**.**0044**
rs1538372|rs4950881|rs10399805|rs6691378|rs946261|rs946262	5-6-7-8-9-10	A-G-G-G-G-A	0.132		11.11	0.03
rs1538372|rs4950881|rs10399805|rs6691378|rs946261|rs946262	5-6-7-8-9-10	A-A-G-G-G-G	0.039		12.12	0.035
rs10399805|rs6691378|rs946261|rs946262|rs116415868|rs10920579	7-8-9-10-11-12	G-G-G-G-G-G	0.055		4.83	0.034

137 samples (16 males and 111 females) were analyzed. Mean age = 13.48. Regression analysis was used. Only *P* values < 0.05 are shown. *P* values < 0.017 (Bonferroni correction threshold) are in bold.

**Table 7:** Haplotype association analyses of plasma YKL-40 levels in endophenotype 2.

SNPs IDs	SNPs	Haplotypes	Freq	Coeff	Std. Error	Pr(>|t|)
rs880633|rs1538372|rs4950881|rs10399805|rs6691378|rs946261	4-5-6-7-8-9	A-A-G-G-G-G	0.174	−13.53	2.99	**9**.**9 × 10**^**−6**^
rs4950881|rs10399805|rs6691378|rs946261|rs946262|rs116415868	6-7-8-9-10-11	A-G-G-G-A-G	0.043	8.31	3.38	**0**.**0146**
rs10399805|rs6691378|rs946261|rs946262|rs116415868|rs10920579	7-8-9-10-11-12	G-G-G-A-G-G	0.045	8.85	3.25	**0**.**0071**
rs10399805|rs6691378|rs946261|rs946262|rs116415868|rs10920579	7-8-9-10-11-12	G-G-A-G-G-G	0.544	34.03	11.05	**0**.**0024**

222 samples (25 males and 197 females) were analyzed. Mean age = 13.78. Regression analysis was used. Only *P* values < 0.05 are shown. *P* values < 0.017 (Bonferroni correction threshold) are in bold.

**Table 8:** Haplotype association analyses of plasma YKL-40 levels in endophenotype 3.

SNP IDs	SNPs	Haplotype	Freq	Coeff	Std. Error	Pr(>|t|)
rs880633|rs1538372|rs4950881|rs10399805|rs6691378|rs946261	4-5-6-7-8-9	A-A-G-G-G-G	0.141	−19.14	8.80	0.031
rs4950881|rs10399805|rs6691378|rs946261|rs946262|rs116415868	6-7-8-9-10-11	G-G-G-G-A-G	0.133	−11.42	3.47	**0**.**0011**
rs10399805|rs6691378|rs946261|rs946262|rs116415868|rs10920579	7-8-9-10-11-12	G-G-G-A-G-G	0.19	7.94	3.86	**0**.**041**
rs55700740|rs7542294|rs946259|rs880633|rs1538372|rs4950881	1-2-3-4-5-6	C-G-G-A-A-G	0.241	−6.66	2.30	**0**.**0041**
rs880633|rs1538372|rs4950881|rs10399805|rs6691378|rs946261	4-5-6-7-8-9	A-G-A-G-G-A	0.017	−36.07	8.03	**1**.**0 × 10**−^**5**^
rs880633|rs1538372|rs4950881|rs10399805|rs6691378|rs946261	4-5-6-7-8-9	G-G-A-G-G-A	0.480	36	7.87	**7**.**6 × 10**^**−6**^
rs4950881|rs10399805|rs6691378|rs946261|rs946262|rs116415868	6-7-8-9-10-11	A-G-G-G-A-G	0.038	9.41	3.89	**0**.**016**
rs10399805|rs6691378|rs946261|rs946262|rs116415868|rs10920579	7-8-9-10-11-12	A-A-G-G-G-G	0.134	−12.53	3.48	**0**.**0004**

266 samples (54 males and 212 females) were analyzed. Mean age = 13.69. Regression analysis was used. Only *P* values < 0.05 are shown. *P* values < 0.017 (Bonferroni correction threshold) are in bold.

**Table 9:** Haplotype association analyses of plasma YKL-40 levels in females.

SNPs IDs	SNPs	Haplotypes	Freq	Coeff	Std. Error	Pr(>|t|)
rs55700740|rs7542294|rs946259|rs880633|rs1538372|rs4950881	1-2-3-4--5-6	C-G-G-A-A-A	0.078		2.44	0.021
rs946259|rs880633|rs1538372|rs4950881|rs10399805|rs6691378	3-4-5-6-7-8	G-A-A-A-G-G	0.092		2.30	0.035
rs4950881|rs10399805|rs6691378|rs946261|rs946262|rs116415868	6-7-8-9-10-11	A-G-G-G-A-G	0.040	6.813	2.15	**0**.**0016**
rs10399805|rs6691378|rs946261|rs946262|rs116415868|rs10920579	7-8-9-10-11-12	G-G-G-A-G-G	0.029	7.902	2.08	**0**.**0002**
rs1538372|rs4950881|rs10399805|rs6691378|rs946261|rs946262	5-6-7-8-9-10	G-A-A-A-G-G	0.142	−5.755	2.14	**0**.**0073**
rs4950881|rs10399805|rs6691378|rs946261|rs946262|rs116415868	6--7-8-9-10-11	A-A-A-G-G-G	0.146	−5.563	2.23	**0**.**0129**
rs10399805|rs6691378|rs946261|rs946262|rs116415868|rs10920579	7-8-9-10-11-12	A-A-G-G-G-G	0.146	−8.425	1.78	**2**.**8 × 10**−^**6**^
rs946259|rs880633|rs1538372|rs4950881|rs10399805|rs6691378	3-4-5-6-7-8	A-G-G-A-G-G	0.491		2.27	0.033
rs880633|rs1538372|rs4950881|rs10399805|rs6691378|rs946261	4-5-6-7-8-9	A-A-G-G-G-A	0.088	6.003	2.13	**0**.**0049**
rs880633|rs1538372|rs4950881|rs10399805|rs6691378|rs946261	4-5-6-7-8-9	A-A-G-G-G-G	0.153	−10.08	1.90	**1**.**6 × 10**−^**7**^

639 samples (534 cases and 105 controls) were analyzed. Mean age = 13.43. Regression analysis was used. Only *P* values < 0.05 are shown. *P* values < 0.017 (Bonferroni correction threshold) are in bold.

**Table 10:** Haplotype association analyses of plasma YKL-40 levels in males.

SNPs	SNPs	Haplotypes	Freq	Coeff	Std. Error	Pr(>|t|)
rs55700740|rs7542294|rs946259|rs880633|rs1538372|rs4950881	1-2-3-4-5-6	C-G-G-A-A-G	0.242	−6.98	2.86	**0**.**016**
rs7542294|rs946259|rs880633|rs1538372|rs4950881|rs10399805	2-3-4-5-6-7	G-G-A-A-G-G	0.242		2.90	0.021
rs946259|rs880633|rs1538372|rs4950881|rs10399805|rs6691378	3-4-5-6-7-8	G-A-A-G-G-G	0.238		2.89	0.049
rs880633|rs1538372|rs4950881|rs10399805|rs6691378|rs946261	4-5-6-7-8-9	A-A-G-G-G-G	0.143	−9.01	2.88	**0**.**0021**

186 Samples (97 cases and 89 controls) were analyzed. Mean age = 13.20. Only *P* values < 0.05 are shown. *P* values < 0.017 (Bonferroni correction threshold) are in bold.

### Functional assessment of the role of YKL-40 in AIS pathogenesis

**Figure 3:**
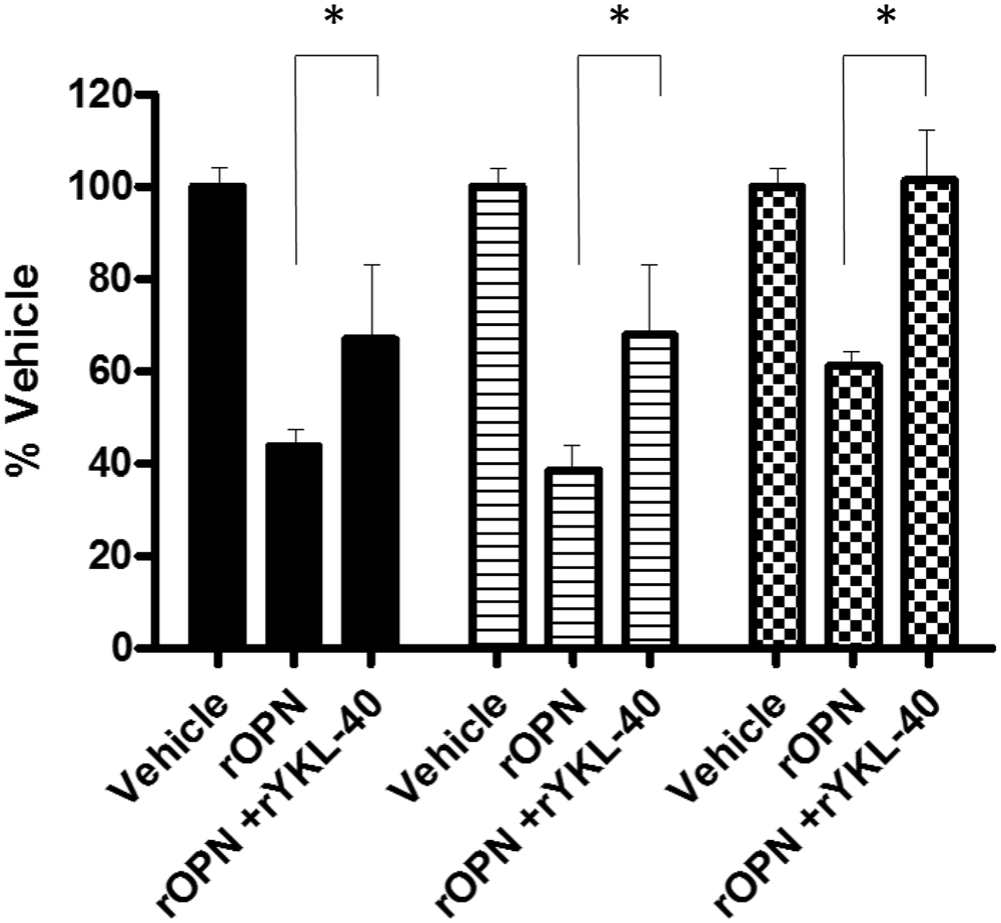
YKL-40 rescues Gi-coupled receptor signalling defect induced by rOPN. Primary osteoblasts obtained from three scoliotic patients were pre-treated with purified rOPN (0.5 µg/ml) with and without rYKL-40 (0.5 µg/ml) for 18 h prior to the stimulation with 10 µM of oxymethazolin. Error bars show SEM of independent experiments performed three times in duplicate. Data represent the percentage of the maximum impedance measured by CDS assay and were normalised to the response achieved in the absence of rOPN (vehicle only). *P < 0.01 based on one-way ANOVA followed by in post-hoc test of Dunnett.

We previously demonstrated the occurrence of a differential Gi-coupled receptor signalling dysfunction in primary osteoblasts and other cell types obtained from AIS patients that led to the identification of three biological endophenotypes associated with AIS as measured by CDS assay^8,9^. To examine the possible functional impact of increased plasma YKL-40 levels, the primary osteoblasts from three scoliotic patients were screened for their responses to oxymetazoline (10 µM), a GiPCR selective agonist activating α1-adrenergic receptor normally coupled to Gi proteins as cellular readout. In agreement with our previous results^9^, exposure to recombinant osteopontin (rOPN) induced a reduction of α1-adrenergic receptor signalling while treatment with purified YKL-40 rescued partially or completely the signalling dysfunction induced by rOPN, suggesting that elevation of YKL-40 could attenuate scoliosis severity (Fig. 3).

## Discussion

To the best of our knowledge, the present study is the first to show a significant association between plasma YKL-40 levels, SNPs regulating *CHI3L1* gene expression and reduced susceptibility to the development of severe spinal deformities in the context of AIS. We showed that AIS patients classified in the non-severe group, at skeletal maturity (Cobb angle 10°–39°), exhibited significant higher plasma YKL-40 levels than controls. We further identified significant associations of multiple *CHI3L1* SNPs and their haplotypes with plasma YKL-40 levels and scoliosis severity. Furthermore, classification of AIS patients as a function of their biological endophenotype revealed that males classified in FG1 endophenotype showed significantly higher plasma YKL-40 levels than controls and AIS patients classified in the two other AIS endophenotypes. This is consistent with the fact that it is also widely known that males are less likely to develop severe forms of the disease when compared to females and we demonstrated previously that AIS patients classified in FG1 endophenotype are less likely to develop a severe spinal deformity when compared to the other endophenotypes^18^. This could be explained in part by the work of Aziz et al. showing an elevation of circulating YKL-40 levels when testosterone levels are increased^26^. Besides, testosterone, decreased circulating ghrelin levels in AIS patients exhibiting a non-progressive scoliosis^20,21^ further support our results obtained with AIS patients classified in the endophenotype FG1 who are less likely to develop a severe scoliosis when compared to AIS patients classified in the other two endophenotypes^18^. Additional studies will be required to characterize the mechanism underlying the regulatory effect of ghrelin on YKL-40 secretion and/or expression in AIS and other conditions.

The previous studies of SNPs regulating circulating YKL-40 levels have demonstrated that genetic variations of the *CHI3L1* gene have an impact on plasma YKL-40 levels, both in healthy subjects as well as in patients suffering diseases from asthma^22^ to rheumatoid arthritis^23^. Indeed, eight of the 12 studied SNPs were associated with plasma YKL-40 levels in our AIS patients while only two of them were associated with YKL-40 plasma levels in healthy controls. The significance of such difference is unclear but in part could be explained by the fact that sample size in controls are smaller than that in cases. Interestingly, the same eight SNPs showed significant associations with YKL-40 plasma levels in the non-severe scoliosis cases. Most of those SNPs have been reported in previous studies to be associated with YKL-40 levels and/or *CHI3L1* expression^14,15,22–25^.

Despite several genome-wide association studies for AIS, none of them reported a signal in/or around the *CHI3L1* gene. However, it should be noted that these studies and our analysis are conceptually very different by design. The associations that we found concerning SNPs and AIS patients were detected owing to the use of more homogenous AIS subgroups determined by our biological endophenotype stratification method^8,9,18^ contrasting with the classical approach using cases vs controls or with the levels of YKL-40 of the different sub-classifications of patients. Genetic studies using intermediate quantitative traits such as biomarkers, or endophenotypes, benefit from increased statistical power to identify variants that may not pass the stringent multiple test correction in case-control studies.

Our study strongly indicates that YKL-40 acts as a protective factor against the progression of spinal deformities in the context of AIS, given its elevation in the non-severe scoliosis group (Table 1). This finding contrasts with a previous study showing an elevation of YKL-40 levels also known as chondrex or HC gp-39, in the cerebrospinal fluid of adult patients suffering of degenerative spine diseases^17^. Indeed, the work of Tsuji et al. showed that the concentration of YKL-40 was more elevated in the degenerative spine disease group with values of 245.3 ± 107.2 ng/ml in cervical myelopathy, 143.2 ± 53.6 ng/ml in lumbar disc herniation, 241.5 ± 77.2 ng/ml in lumbar canal stenosis. The authors suggested that increased YKL-40 concentrations in cerebrospinal fluid resulted in damage or stress to the neural/cartilage structure, and that it could be a new marker for spine diseases^17^. Interestingly, they showed that YKL-40 levels in patients with scoliosis (71.4 ± 33.9 ng/ml) was significantly lower (*P* < 0.001) when compared to other spine diseases or even the control group (113.8 ± 48.3 ng/ml), which is in agreement with our data. Collectively, our data strongly suggest that elevation of YKL-40 levels in idiopathic scoliosis and degenerative scoliosis proceeds through distinct signal-transduction pathways. The identity of cellular receptors mediating the biological effects of YKL-40 in scoliosis remains unknown. To determine a possible causal relationship, we performed *in vitro* functional studies showing that addition of recombinant YKL-40 proteins was sufficient to rescue Gi-coupled receptor signalling defect observed with primary osteoblasts derived from AIS patients. Our functional *in vitro* analysis strongly suggests that elevation of YKL-40 could reduce the severity of scoliosis by interfering with Gi-coupled receptor signalling dysfunction induced by OPN in AIS^9^. We and other groups have reported the role of OPN in scoliosis development in humans and different animal models^27–30^. It remains unclear at the molecular level how YKL-40 is counteracting the effect of OPN in AIS patients and further studies are warranted to determine this mechanism.

In the present study, we acknowledge some limitations. The relatively small sample size of disease progressors (severe scoliosis cases) in each biological endophenotype and the cross-sectional design should be mentioned first. Longitudinal assessment of circulating YKL-40 levels should be considered in combination with the measurement of other biochemical markers such as ghrelin in AIS to better characterize their interplay during puberty and disease progression, including their validation in independent replication cohorts. Finally, the molecular mechanism by which YKL-40 rescues the Gi-coupled receptor signalling dysfunction mediated by OPN in AIS remains to be characterized and represents an unexplored frontier in the field of scoliosis.

In summary, we found a positive correlation of plasma YKL-40 levels with non-severe form of scoliosis as well as with male patients classified in AIS endophenotype FG1, contrasting with patients classified in FG2 endophenotype, who are more prone to develop a severe scoliosis. A negative correlation was observed between circulating ghrelin levels and plasma YKL-40 levels in AIS patients classified in FG1 endophenotype but not in the other two endophenotypes. We also found strong associations of several SNPs and haplotypes of the *CHI3L1* gene with plasma YKL-40 levels and the risk of developing a severe spinal deformity.

## Materials and Methods

### Study populations

A total of 804 French-Canadian AIS patients and 239 age- and sex-matched healthy controls were enrolled between January 2008 and December 2012 in three pediatric spine centers in Montreal and surrounding schools (Table 1 and Supplementary Table 1). All participants are residents of Quebec and of European descent. Each AIS patient was clinically examined by an orthopedic surgeon at the participating hospitals. Full medical history of each participant was collected to assess for other conditions including YKL-40 related diseases (e.g. asthma). We found no other disease at the time of sample collection. All healthy control subjects were screened by an orthopedic surgeon using the Adam’s forward-bending test with a scoliometer. Any children with an apparent spinal curvature or family history of scoliosis were excluded from the control cohort. Ancestral and relatedness testing were performed by applying respectively EIGENSTRAT (Principal Component Analysis or PCA, analysis of self-reported ethnicity) and PLINK identity-by-descent (IBD). Self-reported French-Canadian individuals falling outside the main core cluster were removed from further analyses. Another analysis was performed on the main core cluster to look for any remaining population substructures. Using the IBD approach, ancestral outliers and related samples (pi_hat > 0.1875) were removed prior SNP analyses. Upon classification of the patients based on their spinal deformity severity, at skeletal maturity, 227 AIS patients were considered as non-severe cases (Cobb angle 10°–39°) at the time of measuring the YKL-40 levels, while 132 patients were considered as severe cases (Cobb angle ≥ 40°).

### Ethics statement

Informed written consent was obtained from the parents or their legal guardians of all participants, and minors gave their assent. The study was approved by the Institutional Review Board of Sainte-Justine University Hospital, The Montreal Children’s Hospital, The Shriners Hospital for Children and McGill University as well as by The Affluent School Board and The English School Board of Montreal. All aspects of this research were performed in accordance with the relevant guidelines and regulations.

### Measuring plasma YKL-40 and Ghrelin levels

Peripheral blood samples were collected in EDTA-treated tubes and then centrifuged. Plasma samples were collected, aliquoted and stored at −80 °C until thawed and analyzed. The concentrations of plasma YKL-40 was measured by enzyme-linked immunosorbent assay kit (Quidel, San Diego, CA, USA) according to the protocol provided by the manufacturer. The plasma YKL-40 levels were measured in 728 patients and 216 healthy controls. Unacylated ghrelin was measured in the plasma of a subgroup of 29 AIS patient and 9 matched healthy control subjects by an EIA kit (Cayman Chemicals, Ann Arbor, MI, USA) according to the manufacturer’s specifications. Both assays were performed in duplicate and the mean values were used for the subsequent analyses. The optical density was measured at 450 nm using DTX880 microplate reader (Beckman Coulter, Brea, California, USA).

### Genotyping of SNPs in the *CHI3L1* gene and promoter

Genomic DNA samples were derived from the peripheral blood of the subjects using PureLink® Genomic DNA kit (Thermo Fisher Scientific, Waltham, Massachusetts, USA). Among the cohort, 667 AIS patients and 170 controls were genotyped by the Illumina Human Omni 2.5 M Bead Chip, as part of a study previously conducted by our team at the McGill University and Genome Quebec Innovation Centre^31^. We chose a total of 12 SNPs in the *CHI3L1* gene region due to the fact that their genotypes were already available for most of the cohort. Therefore, these 12 SNPs were also genotyped in a second small cohort, i.e., 137 AIS patients and 51 controls, using multiplex polymerase chain reaction (PCR) at the McGill University and Genome Quebec Innovation. The standard procedures with 20 ng of template genomic DNA and HotStarTaq DNA polymerase enzyme (QIAGEN) were used. The PCR reactions were run on the QIAxcel (QIAGEN) to assess the amplification, followed by single base extension using iPlex Thermo Sequenase. Genotypes were determined by MALDI-TOF mass-spectrometry and the data were analyzed using Mass ARRAY Typer Analyser software.

### Sanger sequencing

Sanger sequencing was performed at the Genome Quebec Innovation Centre at McGill University on a limited subgroup of the AIS patients (n = 7) producing very high circulating YKL-40 levels (>100 ng/ml) and considered as non-severely affected (Supplementary Table 3). The primers were designed using the program Primer3. Sanger sequence chromatograms were analyzed using Mutation Surveyor (Soft Genetics, Inc.).

### Cellular dielectric spectroscopy (CDS) assay

The AIS patient biological endophenotypes were generated from primary osteoblasts or peripheral blood mononuclear cells using cellular dielectric spectroscopy as previously described^8,9^. Through this classification, 145, 257, and 301 patients were classified into the first (FG1), second (FG2), and third (FG3) biological endophenotypes, respectively (Table 1). Functional effects of YKL-40 were measured by a CDS assay as previously described^8,9^. In brief, the primary osteoblasts obtained from bone fragments from AIS patients and control subjects (trauma cases) were seeded into the CellKey^TM^ standard 96-well microplate at a density of 10 × 10^4^ cells per well and incubated in standard conditions (37 °C/5% CO_2_) with 0.5 µg/ml of purified rOPN or the vehicle (saline buffer) for 18 h prior to stimulation. After overnight incubation, cells were directly stimulated with oxymetazoline (10 µM) (Tocris Chemical Co. St. Louis, MO, USA), a specific ligand activating α1-adrenergic receptor normally coupled to Gi proteins. The same test was performed for the cells with and without treatment with recombinant YKL-40 (rYKL-40) to assess its effect.

### Phenotypic analyses

To compare patients and controls and compare among different sub-classifications of patients and controls, an ANOVA test was used with the log-transformed plasma YKL-40 level as the dependent variable and the phenotype and sex as independent variables, with age as covariate. *P* value (two-sided) < 0.05 was considered statistically significant.

### Individual SNP association analyses

The allele frequency of each SNP was calculated separately for each endophenotype sub-classification of the patients and controls. Individual SNP association analyses were performed by comparing the allele frequencies of each SNP between each endophenotype pair of the patients and between patients and controls. The significance was calculated using Fisher’s exact test (two-sided). The software SPSS v.23 was used for these statistical analyses. The quantitative association analysis of the plasma YKL-40 levels with each SNP was performed using the ‘qassoc’ option in PLINK v1.09^32^. The presented *P value*s have been corrected for multiple comparisons using Bonferroni correction.

### Haplotype association analyses

The linkage disequilibrium blocks of the 12 SNPs were estimated based on the genotype data using Haploview^33^. The haplotypes were inferred using UNPHASED^34^ with a window of up to six SNPs. Association analyses were carried out between the inferred haplotypes and the YKL-40 level using an in-house R program. Specifically, a linear regression model was performed for the haplotype associations with YKL-40 levels. The association analyses were also performed based on various subsets of the samples, such as males, females, and endophenotypes. The subgroups with no more than three samples, and the haplotypes with frequency < 0.01 were removed from the analyses. To correct for multiple testing, the experiment-wise significance threshold *P* value was calculated based on the total number of estimated independent linkage disequilibrium blocks. In this study, as no more than three linkage disequilibrium blocks were observed among the 12 SNPs (Fig. 2), three was used as the number of independent tests. Significant associations were reported only when the original *P* value was <0.0167 (corresponding to a corrected *P* value < 0.05).

## Supplementary information

Association of Circulating YKL-40 Levels and CHI3L1 Variants with the Risk of Spinal Deformity Progression in Adolescent Idiopathic Scoliosis
