# Supplementary material for: Association of Circulating YKL-40 Levels and CHI3L1 Variants with the Risk of Spinal Deformity Progression in Adolescent Idiopathic Scoliosis
Source: Sci Rep. 2019 Apr 5;9:5712. doi: 10.1038/s41598-019-41191-4 (PMC6450973; doi:10.1038/s41598-019-41191-4)
Supplement: Supplementary file 1 — Association of Circulating YKL-40 Levels and CHI3L1 Variants with the Risk of Spinal Deformity Progression in Adolescent Idiopathic Scoliosis [file 41598_2019_41191_MOESM1_ESM.docx]

# Association of Circulating YKL-40 Levels and *CHI3L1* Variants with the Risk of Spinal Deformity Progression in Adolescent Idiopathic Scoliosis

Dina Nada^1,2^, Cédric Julien^1^, Pierre H. Rompré^3^, Marie-Yvonne Akoume^1^, Kristen F. Gorman^1,4^ Mark E. Samuels^5,6^, Emile Levy^5,7^, Jason Kost^8^, Dawei Li^8,9^, Alain Moreau^1,2,10,11,*^

^1^ Viscogliosi Laboratory in Molecular Genetics of Musculoskeletal Diseases, Sainte-Justine University Hospital, Research Center, Montreal, QC, Canada; ^2^ Program of Biomedical Sciences, Faculty of Medicine, Université de Montréal, Montreal, QC, Canada; ^3^ Faculty of Dentistry, Université de Montréal, Montreal, QC, Canada; ^4^ Department of Biological Sciences, California State University, Chico, CA, USA; ^5^ Sainte-Justine University Hospital Research Center, Montreal, QC, Canada; ^6^ Department of Medicine, Faculty of Medicine, Université de Montréal, Montreal, QC, Canada; ^7^ Department of Nutrition, Faculty of Medicine, Université de Montréal, Montreal, QC, Canada; ^8^ Department of Microbiology and Molecular Genetics, University of Vermont, Burlington, Vermont, USA; ^9^ Neuroscience, Behavior, and Health Initiative, University of Vermont, Burlington, Vermont, USA; ^10^ Department of Biochemistry and Molecular Medicine, Faculty of Medicine, Université de Montréal, Montreal, QC, Canada; ^11^ Department of Stomatology, Faculty of Dentistry, Université de Montréal, Montreal, QC, Canada.

Correspondence and requests for materials should be addressed to A.M. (email: [alain.moreau@recherche-ste-justine.qc.ca](mailto:alain.moreau@recherche-ste-justine.qc.ca)).

# Supplementary Information

**Materials and Methods**

**Isolation and classification of patient osteoblasts.** Osteoblasts were derived from bone specimens removed from affected vertebrae (number varied from T3 to L4), as a part of correctional surgery for severe scoliosis (AIS cases) and from trauma cases (controls). Under sterile conditions, bone was fragmented with a bone cutter, then incubated in 1x Dulbecco’s Modification Eagle’s Medium (αDMEM) (Wisent Inc, Montreal QC, Canada) supplemented with 10% HyClone Fetal Bovine Serum (FBS) (Thermo Fischer Scientific, Logan UT, USA) and 1% antibiotic (Invitrogen), at 37℃ with 5% CO_2_ until confluent (10-14 days). At confluence, osteoblasts were isolated by trypsinization and frozen in liquid nitrogen. Patient endophenotypes were classified from osteoblast cultures using cellular dielectric spectroscopy (CDS), as previously described in Akoume, et al., 2010 [1]; Akoume, et al., 2013 [2].

**Extraction of RNA.** Total RNA was extracted using Trizol (Invitrogen, CA, USA) according to manufacturer’s instructions. After washing with 70% Ethanol, and dried at RT, RNA was resuspended in 50µl RNase free water (Qiagen, Canada). An aliquot of total RNA diluted in RNase free water was used for quantity and integrity checking (using NanoDrop Version 3.7.1), and the remaining sample was stored at −80^◦^C until gene expression analysis. All samples maintained a 28S/18S rRNA ratio of 1.5 or greater.

**Microarray.** Genome-wide expression patterns were assessed on microarrays performed in triplicate using the Affymetrix, GeneChip® Human Exon 1.0 ST array, at Centre d’Innovation de Génome Québec, Montréal. All protocols were conducted as described in Affymetrix GeneChip Expression Analysis technical manuals. Analysis of microarray data was conducted using GeneSifter software (www.genesifter.net/web/DC). Raw data were normalized using Robust Multi-array Average (RMA) [3], and these derived values were log2-transformed. Data was filtered using a Kruskal-Wallis test followed by a false recovery rate (FDR)-correction according to Benjamini and Hochberg [4], and a threshold of at least a 3-fold differential expression. Probes were considered significant if their FDR-corrected p value was ≤ 0.05. This filtered dataset was explored using clustering algorithms and candidate genes were selected for single-gene analysis via real time quantitative PCR.

**Quantitative Real-Time PCR.** All materials and methods followed MIQE (Minimum Information for Publication of Quantitative Real-Time PCR Experiments) Guidelines [5]. CHI3L1 gene selected from the filtered microarray dataset was further validated by real time quantitative PCR (RT-qPCR) using PerfeCTa SYBR Green SuperMix (Quanta Biosciences) on a 7900HT Real-Time PCR system (Applied Biosystems), according to manufacturer’s protocols. CHI3L1 primers were: Forward primer: 5’-CAGGAAAGCGTCAAAAGCAAGGTG-3’and Reverse primer: 5’- GAGTGCATCCTTGATGGCATTGGT - 3’. For each subject, 1µg of mRNA was reverse transcribed into cDNA using Thermoscript RT-PCR system (Invitrogen CA, USA), according to the manufacturer’s protocol. A 1 in 10 dilution of cDNA was used for RT-qPCR. Each cDNA sample was loaded in triplicate for every gene tested, on an optical 384 reaction plate, and beta-actin was used as an endogenous control for normalizing gene expression, based on Stephens et al., 2011 [6]. The fold-change in gene expression was determined by the program RQ manager [7]. RQ values were log_2_ transformed, and the Levene test checked for equal variances between groups. If variances were equal we used either an ANOVA or a T-test, if not equal we used the Wilcoxon/Kruskal-Wallis test to examine whether average expression levels were different among clusters. Statistical analyses were performed using JMP 9 software (SAS Institute Inc. 2012).

**References**

1. Akoume MY, Azeddine B, Turgeon I, et al. (2010) Cell-based screening test for idiopathic scoliosis using cellular dielectric spectroscopy. Spine (Phila Pa 1976) 35(13): E601-8.

2. Akoume MY, Franco A, Moreau A. (2013) Cell-based Assay Protocol for the Prognostic Prediction of Idiopathic Scoliosis Using Cellular Dielectric Spectroscopy. J. Vis. Exp. 80: e50768.

3. Irizarry RA, Bolstad BM, Collin F, et al. (2003) Summaries of Affymetrix GeneChip probe level data. Nucleic Acids Res. 31(4): e15.

4. Hu JX, Zhao H, Zhou HH. (2010) False discovery rate control with groups. Journal of the American Statistical Association 104 (491): 1215-1227.

5. Bustin SA, Benes V, Garson JA, et al. (2009) The MIQE guidelines: minimum information for publication of quantitative real-time PCR experiments. Clin Chem. 55(4): 611-22.

6. Stephens AS, Stephens SR, Morrison NA. (2011) Internal control genes for quantitative RT-PCR expression analysis in mouse osteoblasts, osteoclasts and macrophages. BMC Res Notes. 4:410.

7. Livak KJ, and Schmittgen TD. (2001) Analysis of Relative Gene Expression Data Using Real-Time Quantitative PCR and the 2^–ΔΔCT^ Method. Methods 25:402–408.

**Supplementary Table 1. Demographic and clinical data for the replication cohort genotyped using multiplex PCR**

|  | N | Age (years) | Cobb Angle (°) |
| --- | --- | --- | --- |
| Healthy Controls | 51 | 12.2 ± 3.8  (3.2-18.3) | - |
| Female Controls | 30 | 12.4 ± 4.0  (5.0-18.3) | - |
| Male Controls | 21 | 11.8 ± 3.4  (3.2-15.9) | - |
| All AIS Patients | 137 | 13.7 ± 2.1  (8.1-18.6) | 27 ± 16  (10-81) |
| Female AIS Patients | 117 | 13.6 ± 2.1  (8.1-18.6) | 28 ± 16  (10-81) |
| Male AIS Patients | 20 | 14.2 ± 1.8  (11.3-17.9) | 21 ± 11  (10-54) |

**Supplementary Table 2. Demographic and clinical data for a subset of AIS patients and controls subjects tested for unacylated ghrelin**

|  | N | Age (years) | Cobb Angle (°) | YKL-40 Levels (ng/ml) | Ghrelin Levels (pg/ml) |
| --- | --- | --- | --- | --- | --- |
| Healthy Controls | 9 | 14.3 ± 1.7  (10.9-16.7) | - | 19.3 ± 5.8  (8.1-31.1) | 162.8 ± 63.9  (82.4-292.5) |
| All AIS Patients | 29 | 14.9 ± 1.8  (10.5-18.9) | 41 ± 17  (19-75) | 22.7 ±9.2  (7.0-38.9) | 123.3 ± 115.2  (36.1-484.3) |
| FG1 AIS Patients | 9 | 15.3 ± 1.2  (13.5-16.6) | 39 ± 12  (19-52) | 22.9 ± 9.7  (8.8-37.4) | 99.9 ± 44.9  (48.7-192.9) |
| FG2 AIS Patients | 10 | 14.3 ± 2.5  (10.5-18.9) | 58 ± 10  (45-75) | 24.5 ± 9.3  (11.4-38.9) | 147.2 ± 178.0  (36.1-484.3) |
| FG3 AIS Patients | 10 | 15.0 ± 1.2  (13.2-16.6) | 25 ± 7  (19-44) | 20.6 ± 9.4  (7.0-35.9) | 120.4 ± 81.3  (40.9-313.1) |

**Supplementary Table 3. Demographic and clinical data for AIS patients identified as YKL-40 overproducers**

| Patient ID | Sex | Age (years) | Cobb Angle (°) | Curve Type | YKL-40 Levels (ng/ml) | Biological endophenotype |
| --- | --- | --- | --- | --- | --- | --- |
| 165 | F | 10.8 | 26-23 | rTlL | 182.94 | FG2 |
| 336 | F | 14.0 | 9-20 | rTlL | 112.54 | FG3 |
| 497 | F | 17.8 | 30-19 | rTlL | 98.29 | FG3 |
| 572 | M | 10.9 | 11 | rT | 297.93 | FG1 |
| 668 | F | 12.9 | 12-12 | rTlL | 158.30 | FG2 |
| 693 | M | 14.7 | 23-13 | rTlL | 228.23 | FG2 |
| 1590 | F | 9.2 | 21-25-19 | lTrTlL | 109.86 | FG2 |

rTlL: right thoracic and left lumbar; rT: righ thoracic; lTrTlL: left thoracic, right thoracic and left lumbar

**Supplementary Table 4. Comparison between AIS biological endophenotypes for the associations of each of the 12 SNPs**

| SNP | Group1 VS Control | Group2 VS Control | Group3 VS Control | Group1 VS Group2 | Group1 VS Group3 | Group2 VS Group3 |
| --- | --- | --- | --- | --- | --- | --- |
| rs55700740 | P=0.899 | P=0.099 | P=0.437 | P=0.089 | P=0.415 | P=0.509 |
| rs7542294 | P=0.027 | P=0.195 | P=0.248 | P=0.042 | P=0.232 | P=0.606 |
| rs946259 | P=0.918 | P=0.082 | P=0.338 | P=0.108 | P=0.485 | P=0.411 |
| rs880633 | P=0.908 | P=0.084 | P=0.349 | P=0.107 | P=0.486 | P=0.411 |
| rs1538372 | P=0.186 | P=0.133 | P=0.832 | **P=0.001** | P=0.072 | P=0.15 |
| rs4950881 | P=0.368 | P=0.706 | P=0.485 | P=0.099 | P=0.09 | P=0.472 |
| rs10399805 | P=0.058 | P=0.153 | P=0.434 | P=0.028 | P=0.112 | P=0.626 |
| rs6691378 | P=0.027 | P=0.094 | P=0.432 | p=0.017 | P=0.142 | P=0.319 |
| rs946261 | P=0.368 | P=0.014 | P=0.444 | P=0.388 | P=0.95 | P=0.153 |
| rs946262 | P=0.773 | P=0.097 | P=0.672 | P=0.036 | P=0.902 | P=0.025 |
| rs116415868 | P=0.644 | P=0.27 | P=0.08 | P=0.267 | P=0.095 | P=0.315 |
| rs10920579 | P=0.798 | P=0.347 | P=0.729 | P=0.306 | P=0.895 | P=0.451 |

**Supplementary Table 5. Sample sizes for haplotype analyses of plasma YKL-40 levels**

|  | Case | Control | Male | Female | Total |
| --- | --- | --- | --- | --- | --- |
| YKL-40 total | 631 | 194 | 186 | 639 | 825 |
| Male | 97 | 89 | 186 | 0 | 186 |
| Female | 534 | 105 | 0 | 639 | 639 |
| Endophenotype 1 | 127 | 0 | 16 | 111 | 127 |
| Endophenotype 2 | 222 | 0 | 25 | 197 | 222 |
| Endophenotype 3 | 266 | 0 | 54 | 212 | 266 |


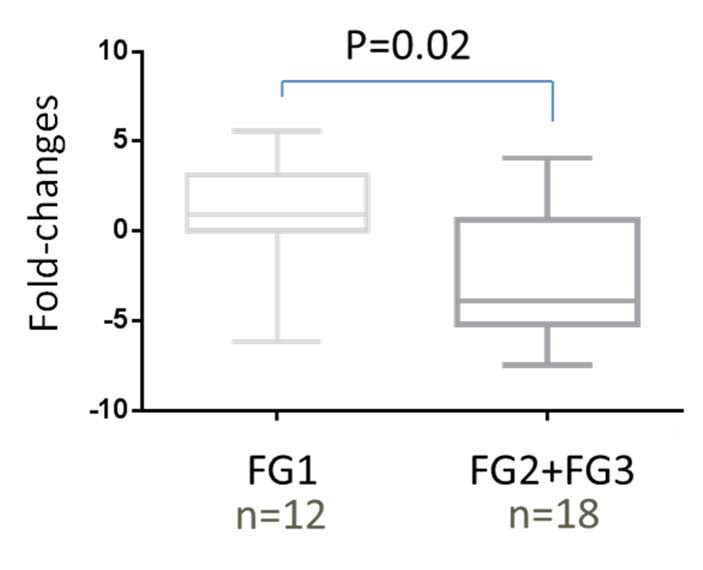


**Supplementary Figure 1. Expression analysis of *CHI3L1* gene in primary human osteoblasts obtained from AIS patients classified in FG1 biological endophenotype vs. AIS patients classified in FG2+FG3 biological endophenotypes.** Statistical analysis was performed with an unpaired T-test (two-tailed *P*-value).
